# Discovery of 2,4-thiazolidinedione-tethered coumarins as novel selective inhibitors for carbonic anhydrase IX and XII isoforms

**DOI:** 10.1080/14756366.2021.2024528

**Published:** 2022-01-07

**Authors:** Wagdy M. Eldehna, Mohammed S. Taghour, Tarfah Al-Warhi, Alessio Nocentini, Mostafa M. Elbadawi, Hazem A. Mahdy, Mohamed A. Abdelrahman, Ohoud J. Alotaibi, Nada Aljaeed, Diaaeldin M. Elimam, Kamyar Afarinkia, Hatem A. Abdel-Aziz, Claudiu T. Supuran

**Affiliations:** a Department of Pharmaceutical Chemistry, Faculty of Pharmacy, Kafrelsheikh University, Kafrelsheikh, Egypt; b Pharmaceutical Medicinal Chemistry & Drug Design Department, Faculty of Pharmacy, Al-Azhar University, Cairo, Egypt; c Department of Chemistry, College of Science, Princess Nourah Bint Abdulrahman University, Riyadh, Saudi Arabia; d Department of NEUROFARBA, Section of Pharmaceutical and Nutraceutical Sciences, University of Florence, Firenze, Italy; e Department of Pharmaceutical Chemistry, Faculty of Pharmacy, Egyptian Russian University, Cairo, Egypt; f Department of Pharmacognosy, Faculty of Pharmacy, Kafrelsheikh University, Kafrelsheikh, Egypt; g School of Molecular and Cellular Biology, Faculty of Biological Sciences, University of Leeds, Leeds, United Kingdom; h Institute of Cancer Therapeutics, University of Bradford, Bradford, United Kingdom; i Department of Applied Organic Chemistry, National Research Center, Giza, Egypt

**Keywords:** Carbonic anhydrase inhibitors, 3-acetylcoumarin, apoptosis induction, anticancer agents, hypoxic tumours

## Abstract

Different 2,4-thiazolidinedione-tethered coumarins **5a–b**, **10a–n** and **11a–d** were synthesised and evaluated for their inhibitory action against the cancer-associated *h*CAs IX and XII, as well as the physiologically dominant *h*CAs I and II to explore their selectivity. Un-substituted phenyl-bearing coumarins **10a**, **10 h**, and 2-thienyl/furyl-bearing coumarins **11a–c** exhibited the best *h*CA IX (K_I_s between 0.48 and 0.93 µM) and *h*CA XII (K_I_s between 0.44 and 1.1 µM) inhibitory actions. Interestingly, none of the coumarins had any inhibitory effect on the off-target *h*CA I and II isoforms. The sub-micromolar compounds from the biochemical assay, coumarins **10a**, **10 h** and **11a–c**, were assessed in an *in vitro* antiproliferative assay, and then the most potent antiproliferative agent **11a** was tested to explore its impact on the cell cycle phases and apoptosis in MCF-7 breast cancer cells to provide more insights into the anticancer activity of these compounds.

## 1. Introduction

Carbonic anhydrases (CAs, EC 4.2.1.1) are ubiquitous metalloenzymes found in all living organisms and are responsible for the catalysis of the biologically crucial reversible hydration of carbon dioxide to bicarbonate and proton. This is a simple but pivotal physiological reaction which is essential for normal and pathological processes such as CO_2_ and pH homeostasis, respiration, gluconeogenesis, calcification, bone resorption, fluid secretion and tumorigenesis [1,2]. CAs are grouped into different families, amongst them α-CAs which are present in all vertebrates and are further sub-classified into fifteen isoforms (herein referred to as human CAs or *h*CAs) that differ by molecular features, expression levels, kinetic properties and cellular distribution in the different tissues [3]. Of note, only twelve *h*CAs are catalytically active (I–IV, VA, VB, VI, VII, IX, XII–XIV) with an active site containing three histidine residues in a triple coordination with a zinc ion [4]. Regarding the subcellular distribution of the catalytically active *h*CAs, they can be categorised into different subsets: cytosolic (I, II, III, VII and XIII), trans-membrane (IV, IX, XII, and XIV), mitochondrial (VA and VB), while VI is secreted in saliva and milk [5]. The over-expressed levels and/or dysfunctions of *h*CAs can lead to many disorders, hence CA inhibitors (CAIs) are utilised for the treatment of glaucoma (targeting *h*CA II, IV and XII), edoema (targeting *h*CA II, IV and XIV), mental disorders (targeting *h*CA II, VII and XIV) and obesity (targeting *h*CA VA and VB) [4,6,7].

It should be stressed that the trans-membranous *h*CA IX and XII are hypoxia-induced tumours-associated isozymes and overexpressed in most cancer cells compared to the normal ones [8]. While the overexpressed *h*CA IX isozyme is mainly linked to cancer poor prognosis and limited to hypoxic tumours, *h*CA XII can be found in some normal tissues like kidney and colon alongside the hypoxic tumours [9,10]. Interestingly, the tumour growth, angiogenesis, proliferation and metastasis are attributed to the overexpressed levels of *h*CA IX and XII suggesting a strategy for targeting of such enzymes as a new approach in cancer chemotherapy [8,11]. In this context, selective inhibition of the tumour-associated *h*CA IX and XII isozymes over the other isoforms, particularly the most prevalent cytosolic *h*CA I and II is highly desirable and will result in cancer treatment with fewer side effects [12].

In view of this, intensive efforts are being conducted for the development of *h*CA IX/XII selective inhibitors as a validated approach for cancer treatment [13–15]. *h*CA IX and XII can be inhibited by different strategies such as coordination to the zinc ion situated in the catalytic active site. Molecules in this class are exemplified by sulfonamide-derived *h*CAIs and their bioisosters. In addition, the occlusion of the catalytic active cleft is explored and this approach has been explored using coumarins as a newly discovered *h*CAIs class [16,17].

**Figure 1.**
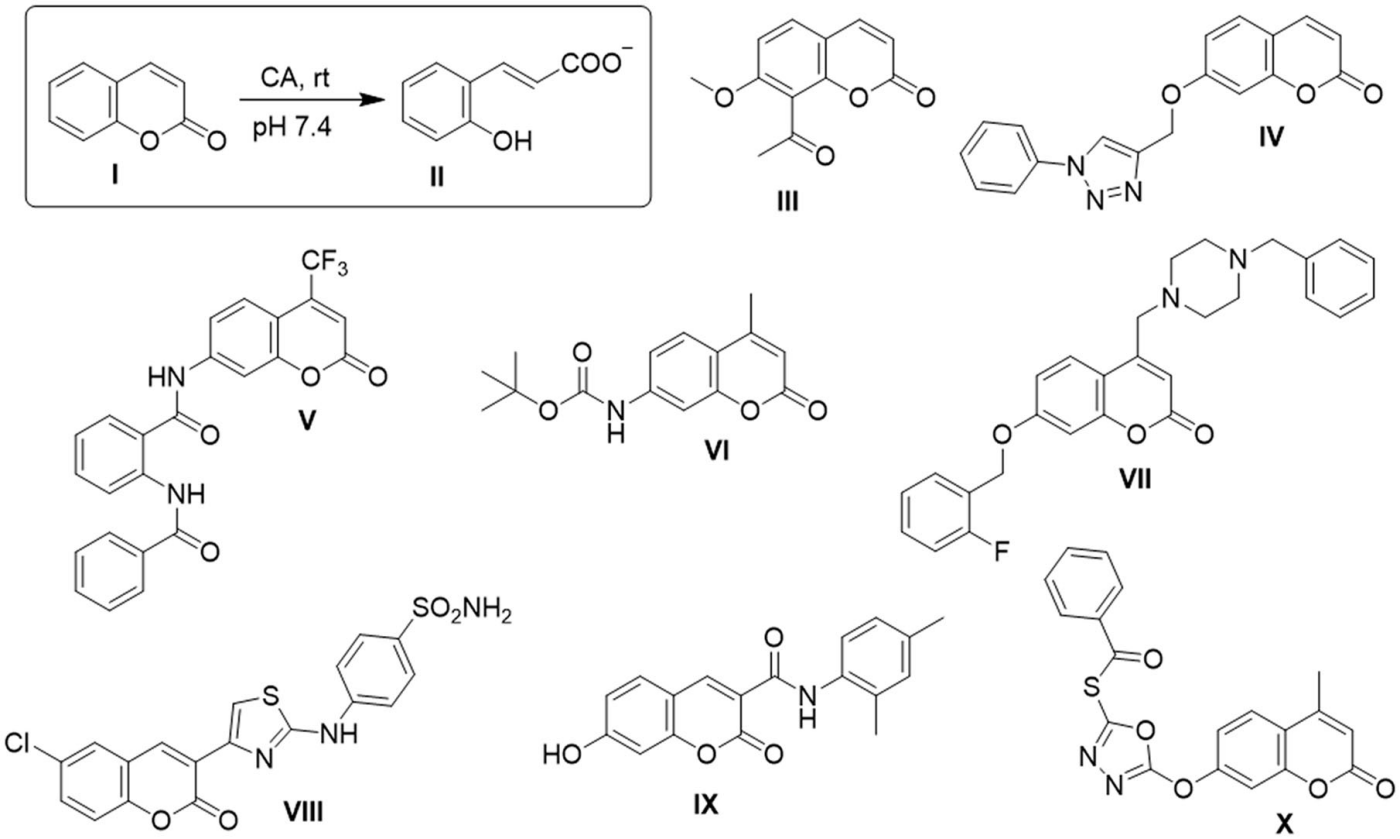
Some reported coumarins acting as selective CA IX/XII inhibitors.

Coumarin **I** is a naturally-derived, privileged heterocyclic scaffold and molecules containing it show numerous biological properties such as inhibition of CK2, EGFR and PI3K-AKT-mTOR signalling. Moreover, coumarins are known to have anticoagulation, monoamine oxidase inhibition, anti-infective, antioxidant, anti-inflammatory and anticancer activities [14,18–20]. Coumarins are recently discovered as a novel class of *h*CAIs with inhibitory mechanism different from the sulfonamide-based inhibitors. Coumarinacts as prodrug undergoing hydrolysis by the esterase activity of CA to yield 2-hydroxycinnamic acid derivative **II** which can bind to the active site cleft occluding its entrance, Figure 1 [15,21]. Since coumarins binding sites are the most heterologous region of the active site between all CAs isoforms, it not surprising that these chemotypes displaying very high selectivity for specific CAs isoforms. Furthermore, the chemical simplicity of coumarins permits the facile incorporation of diverse substituents, leading to generation of a large number of derivatives with interesting biological profiles [22]. Consequently, many ongoing efforts have focussed on developing novel coumarin derivatives as selective *h*CAs IX/XII inhibitors that could be used for cancer therapy. For instance, diverse coumarins **III-X** have been reported as selective *h*CAs IX/XII inhibitors with nanomolar K_i_, Figure 1 [8,10,13,17,21–24].

Inspired by these findings, we prepared a series of 2,4-thiazolidinedione-tethered coumarins, compounds **5a–b**, **10a–n** and **11a–d**, and evaluated their inhibitory action against the cancer-associated *h*CAs IX and XII, and selectivity over inhibition of the physiologically dominant *h*CAs I and II to explore their selectivity in order to the cancer-related isoforms. Moreover, the efficient *h*CAs IX/XII inhibitors **10a**, **10h** and **11a–c** were subjected to *in vitro* antiproliferative assay under hypoxic conditions and most potent antiproliferative agent **11a** was tested to explore its impact on the cell cycle phases and apoptosis in MCF-7 breast cancer cells furnishing more insights on the anticancer activity of such compounds.

## 2. Experimental

### 2.1. Chemistry

#### 2.1.1. General

The NMR spectra were recorded by Bruker 400 MHz spectrometer. ^1^H and ^13 ^C spectra were run at 400 and 100 MHz, respectively, in deuterated dimethylsulphoxide (DMSO-*d_6_*) or deuterated triflouroacetic acid. All coupling constant (*J*) values are given in hertz. IR spectra were recorded with a Bruker FT-IR spectrophotometer. Reaction courses and product mixtures were routinely monitored by thin layer chromatography (TLC) on silica gel precoated F_254_ Merck plates. Unless otherwise mentioned, all reagents and solvents are commercially available and have been used without further purification. Compounds **2** and **3** are previously reported [25,26].

#### 2.1.2. General procedures for synthesis of 3–(2-oxo-2–(2-oxo-2*H*-chromen-3-yl)ethyl)thiazolidine-2,4-dione derivatives (5a–b)

To a stirred solution of 3–(2-bromoacetyl)-2*H*-chromen-2-one **3a** (0.27 g, 1.0 mmol) or 6-bromo-3–(2-bromoacetyl)-2*H*-chromen-2-one **3b** (0.34 g, 1.0 mmol) in DMF (7 ml), thiazolidine-2,4-dione **4** (0.12 g, 1.0 mmol), anhydrous K_2_CO_3_ (0.28 g, 2.0 mmol) and KI (cat.) were added. The reaction mixture was heated on a water bath for 8h. then was poured over crushed ice. The precipitate was filtered, dried, and crystalsized from hot ethanol to give the corresponding key intermediates **5a-b**, respectively.

##### 2.1.2.1. 3–(2-Oxo-2–(2-oxo-2H-chromen-3-yl)ethyl)thiazolidine-2,4-dione 5a

Yellow crystals (yield, 80%); m. p. = 180–181 °C (reported: 161–163 °C [27]); ^1^H NMR (400 MHz, Trifluoroacetic acid-*d_1_*): *δ* 8.79 (s, 1H, H-4 coumarin moiety), 8.01 (d, *J* = 8.0 Hz, 1H, H-5 coumarin moiety), 7.81 (t, *J* = 7.2 Hz, 1H, H-7 coumarin moiety), 7.51 (d, *J* = 8.0 Hz, 1H, H-8 coumarin moiety), 7.46 (t, *J* = 7.6 Hz, 1H, H-6 coumarin moiety), 5.13 (s, 2H, CH_2_, –N–CH_2_–CO–), 4.10 (s, 2H, CH_2_, –S–CH_2_–CO–); ^13 ^C NMR (101 MHz, Trifluoroacetic acid-d_1_) *δ* 189.47, 172.32, 159.16, 155.25, 149.25, 135.73, 131.67, 125.63, 122.60, 118.53, 116.75, 50.43 (–N–CH_2_–), 34.54 (–S–CH_2_–); Anal. Calcd. for C_14_H_9_NO_5_S (303.29): C, 55.44; H, 2.99; N, 4.62; Found: C, 55.68; H, 3.01; N, 4.56.

##### 2.1.2.2. 3–(2-(6-Bromo-2-oxo-2H-chromen-3-yl)-2-oxoethyl)thiazolidine-2,4-dione 5b

Yellow crystals (yield, 75%); m. p.= 239–241 °C (reported: 171–173 °C [27]); ^1^H NMR (400 MHz, DMSO-d_6_) *δ* 8.59 (s, 1H, H-4 coumarin), 8.20 (s, 1H, H-5 coumarin), 7.88 (d, *J* = 8.8 Hz, 1H, H-7 coumarin), 7.44 (d, *J* = 8.8 Hz, 1H, H-8 coumarin), 5.14 (s, 2H, CH_2_), 4.11 (s, 2H, CH_2_); ^13 ^C NMR (101 MHz, TFA-deuterated) *δ* 195.41, 158.46, 154.08, 146.09, 137.04, 132.10, 125.85, 120.52, 118.86, 116.82, 60.22 (–N–CH_2_–), 30.49 (–S–CH_2_–); Anal. Calcd. for C_14_H_8_BrNO_5_S (382.18): C, 44.00; H, 2.11; N, 3.66; Found: C, 43.85; H, 2.12; N, 3.69.

#### 2.1.3. General procedures for preparation of the intermediates 8a–g and 9a–b

To a solution of thiazolidine-2,4-dione **4** (0.12 g, 1 mmol) in glacial acetic acid (5 ml), anhydrous sodium acetate (0.08 g, 1 mmol) and the appropriate aldehyde derivative (**6a–g** and **7a–b**) were added. The resulting reaction mixture was allowed to stir under reflux for 3h. The precipitated solid was collected by filtration while hot, washed with cold ethanol and water, and dried to afford intermediates **8a–g** and **9a–b**.

#### 2.1.4. General procedures for preparation of coumarins 10a–n and 11a–d

The appropriate benzylidine derivative **8a–g** (2 mmol) was added to a hot stirred mixture of 3-(bromoacetyl)coumarin derivatives **3a–b** (2 mmol), K_2_CO_3_ (0.55 g, 4 mmol), KI (2 mmol) in DMF (8 ml), then the resulting mixture was stirred under reflux for 8h. The formed precipitates were collected by filtration, washed with water, dried and recrystalslized from DMF/water to yield the final target coumarin-based CAIs **10a–n** and **11a–d.**

##### 2.1.4.1. 5-Benzylidene-3–(2-oxo-2–(2-oxo-2H-chromen-3-yl)ethyl)thiazolidine-2,4-dione 10a

Grey crystals (yield, 70%); m. p. = 249–251 °C; ^1^H NMR (400 MHz, DMSO-d_6_) *δ* 9.17 (s, 1H, ArH), 8.27 (s, 1H, –CH=), 8.01–7.97 (m, 2H, ArH), 7.70–7.61 (m, 7H, ArH), 5.64 (s, 2H, CH_2_, –N–CH_2_–CO–); ^13 ^C NMR (101 MHz, TFA-deuterated) *δ* 173.27, 172.54, 168.52, 168.28, 153.27, 139.52, 139.52, 137.19, 132.00, 131.79, 131.19, 130.47, 129.01, 126.36, 118.57, 116.70, 115.75, 54.32 (–N–CH_2_–); Anal. Calcd. for C_21_H_13_NO_5_S (391.40): C, 64.44; H, 3.35; N, 3.58; Found: C, 64.71; H, 3.37; N, 3.52 [28].

##### 2.1.4.2. 5–(4-Methylbenzylidene)-3–(2-oxo-2–(2-oxo-2H-chromen-3-yl)ethyl)thiazolidine-2,4-dione 10b

Yellow crystals (yield, 85%); m. p. = 227–229 °C; ^1^H NMR (400 MHz, DMSO-d_6_) *δ* 8.61 (s, 1H, ArH), 8.04 (d, *J* = 8.0 Hz, 1H, ArH), 7.79 (t, *J* = 7.6 Hz, 1H, H-7 ArH), 7.72 (s, 1H, –CH=), 7.51 (d, *J* = 8.0 Hz, 1H, ArH), 7.47 (d, *J* = 8.0 Hz, 2H, ArH), 7.48 (t, *J* = 7.6 Hz, 1H, ArH), 7.33 (d, *J* = 8.0 Hz, 2H, ArH), 5.13 (s, 2H, CH_2_, –N–CH_2_–CO–), 2.41 (s, 3H, CH_3_); Anal. Calcd. for C_22_H_15_NO_5_S (405.42): C, 65.18; H, 3.73; N, 3.45; Found: C, 64.95; H, 3.76; N, 3.51.

##### 2.1.4.3. 5–(4-Methoxybenzylidene)-3–(2-oxo-2–(2-oxo-2H-chromen-3-yl)ethyl)thiazolidine-2,4-dione 10c

Yellow crystals (yield, 85%); m. p. = 257–259 °C; ^1^H NMR (400 MHz, DMSO-d_6_) *δ* 9.16 (s, 1H, ArH), 8.24 (s, 1H, –CH=), 8.00–7.96 (m, 2H, ArH), 7.71 (d, *J* = 8.8 Hz, 2H, ArH), 7.65–7.61 (m, 2H, ArH), 7.23 (d, *J* = 8.8 Hz, 2H, ArH), 5.63 (s, 2H, CH_2_), 4.10 (s, 3H, OCH_3_); ^13 ^C NMR (101 MHz, TFA-deuterated) δ 172.54, 168.38, 162.43, 162.00, 161.56, 161.13, 153.19, 139.03, 137.03, 132.98, 131.07, 126.39, 126.00, 118.52, 118.05, 116.05, 115.71, 112.90, 110.08, 54.90 (OCH_3_), 51.47 (–N–CH_2_–); Anal. Calcd. for C_22_H_15_NO_6_S (421.42): C, 62.70; H, 3.59; N, 3.32; Found: 62.88; H, 3.62; N, 3.26 [28].

##### 2.1.4.4. 5–(2,5-Dimethoxybenzylidene)-3–(2-oxo-2–(2-oxo-2H-chromen-3-yl)ethyl)thiazolidine-2,4-dione 10d

Grey crystals (yield, 82%); m. p. = 238–240 °C; ^1^H NMR (400 MHz, DMSO-d_6_) *δ* 8.87 (s, 1H, ArH), 8.28 (s, 1H, –CH=), 7.76–7.73 (m, 2H, ArH), 7.42–7.39 (m, 2H, ArH), 7.08–6.93 (m, 3H, ArH), 5.36 (s, 2H, CH_2_), 3.84 (s, 3H, OCH_3_), 3.82 (s, 3H, OCH_3_); ^13 ^C NMR (101 MHz, DMSO) *δ* 206.65, 189.23, 167.42, 165.61, 159.17, 153.66, 153.59, 152.95, 149.34, 135.63, 131.61, 129.04, 122.59, 122.52, 122.23, 121.89, 117.80, 116.65, 113.64, 56.31 (OCH_3_), 55.73 (OCH_3_), 50.67 (–N–CH_2_–); Anal. Calcd. for C_23_H_17_NO_7_S (451.45): C, 61.19; H, 3.80; N, 3.10; Found: 60.94; H, 3.83; N, 3.14.

##### 2.1.4.5. 5–(4-Nitrobenzylidene)-3–(2-oxo-2–(2-oxo-2H-chromen-3-yl)ethyl)thiazolidine-2,4-dione 10e

Yellow crystals (yield, 77%); m. p. = 263–265 °C; ^1^H NMR (400 MHz, DMSO-d_6_) *δ* 8.84 (s, 1H, ArH), 8.36 (d, *J* = 8.8 Hz, 2H, ArH), 8.13 (s, 1H, –CH=), 8.00 (d, *J* = 8.4 Hz, 1H, ArH), 7.93 (d, *J* = 8.4 Hz, 2H, ArH), 7.86–7.79 (m, 2H, ArH), 7.44 (t, *J* = 8.0 Hz, 1H, ArH), 5.24 (s, 2H, CH_2_, –N–CH_2_–CO–); ^13 ^C NMR (101 MHz, DMSO) *δ* 189.33, 165.32, 159.25, 155.31, 149.42, 148.23, 139.55, 135.84, 131.73, 131.65, 131.27, 128.47, 125.66, 124.72, 122.55, 118.57, 116.79, 50.99 (–N–CH_2_–); Anal. Calcd. for C_21_H_12_N_2_O_7_S (436.39): C, 57.80; H, 2.77; N, 6.42; Found: C, 58.03; H, 2.75; N, 6.49.

##### 2.1.4.6. 5–(2-Chlorobenzylidene)-3–(2-oxo-2–(2-oxo-2H-chromen-3-yl)ethyl)thiazolidine-2,4-dione 10f

Yellow crystals (yield, 77%); m. p. = 208–211 °C; ^1^H NMR (400 MHz, DMSO-d_6_) *δ* 8.84 (s, 1H, ArH), 8.06 (s, 1H, –CH=), 8.01 (d, *J* = 8.0 Hz, 1H, ArH), 7.84 (d, *J* = 8.0 Hz, 1H, ArH), 7.79 (d, *J* = 8.4 Hz, 1H, ArH), 7.71 (d, *J* = 8.0 Hz, 1H, ArH), 7.66–7.59 (m, 2H, ArH), 7.51 (d, *J* = 8.4 Hz, 1H, ArH), 7.44 (t, *J* = 8.4 Hz, 1H, ArH), 5.23 (s, 2H, CH_2_, –N–CH_2_–CO–); ^13 ^C NMR (101 MHz, DMSO) *δ* 189.39, 167.21, 165.20, 159.24, 149.43, 135.84, 134.17, 133.07, 132.88, 132.04, 131.74, 129.69, 129.24, 125.79, 125.67, 125.19, 122.57, 118.56, 116.79, 55.39 (–N–CH_2_–); Anal. Calcd. for C_21_H_12_ClNO_5_S (425.84): C, 59.23; H, 2.84; N, 3.29; Found: C, 59.43; H, 2.82; N, 3.25.

##### 2.1.4.7. 5–(2-Bromobenzylidene)-3–(2-oxo-2–(2-oxo-2H-chromen-3-yl)ethyl)thiazolidine-2,4-dione 10g

Yellow crystals (yield, 82%); m. p. = 230–232 °C; ^1^H NMR (400 MHz, DMSO-d_6_) *δ* 9.15 (s, 1H, ArH), 8.64 (s, 1H, –CH=), 8.00–7.97 (m, 2H, ArH), 7.74–7.72 (m, 1H, ArH), 7.66–7.63 (m, 3H, ArH), 7.53–7.56 (m, 2H, ArH), 5.65 (s, 2H, CH_2_, –N–CH_2_–CO–); ^13 ^C NMR (101 MHz, TFA-deuterated) *δ* 190.49, 172.12, 167.88, 163.26, 162.48, 162.04, 161.61, 161.17, 155.14, 136.60, 130.43, 121.61, 119.96, 118.49, 118.05, 115.68, 112.86, 110.05, 50.72 (–N–CH_2_–); Anal. Calcd. for C_21_H_12_BrNO_5_S (470.29): C, 53.63; H, 2.57; N, 2.98; Found: C, 53.81; H, 2.58; N, 3.01

##### 2.1.4.8. 5-Benzylidene-3–(2-(6-bromo-2-oxo-2H-chromen-3-yl)-2-oxoethyl)thiazolidine-2,4-dione 10h

Yellow crystals (yield, 78%); m. p. = 252–254 °C; ^1^H NMR (400 MHz, DMSO-d_6_) *δ* 8.60 (s, 1H, ArH), 8.21 (d, *J* = 2.4 Hz, 1H, ArH), 7.87 (d, *J* = 7.6 Hz, 1H, ArH), 7.78 (s, 1H, –CH=), 7.43–7.61 (m, 6H, ArH), 5.14 (s, 2H, CH_2_, –N–CH_2_–CO–); ^13 ^C NMR (101 MHz, DMSO) *δ* 168.61, 168.30, 146.11, 137.06, 134.29, 133.60, 133.42, 133.01, 131.94, 130.81, 130.45, 129.78, 125.88, 124.43, 120.54, 118.88, 117.04, 116.83, 50.83(–N–CH_2_–); Anal. Calcd. for C_21_H_12_BrNO_5_S (470.29): C, 53.63; H, 2.57; N, 2.98; Found: C, 53.78; H, 2.56; N, 3.00.

##### 2.1.4.9. 3–(2-(6-Bromo-2-oxo-2H-chromen-3-yl)-2-oxoethyl)-5–(4-methylbenzylidene)thiazolidine-2,4-dione 10i

Yellow crystals (yield, 86%); m. p. = 224–225 °C; ^1^H NMR (400 MHz, DMSO-d_6_) *δ* 8.60 (s, 1H, ArH), 8.20 (s, 1H, ArH), 7.86 (d, *J* = 8.8 Hz, 1H, ArH), 7.78 (s, 1H, –CH=), 7.47 (d, *J* = 8.0 Hz, 2H, ArH), 7.42 (d, *J* = 8.8 Hz, 1H, ArH), 7.33 (d, *J* = 8.0 Hz, 2H, ArH), 5.13 (s, 2H, –N–CH_2_–CO–), 2.35 (s, 3H, CH_3_); Anal. Calcd. for C_22_H_14_BrNO_5_S (484.32): C, 54.56; H, 2.91; N, 2.89; Found: C, 54.75; H, 2.89; N, 2.90.

##### 2.1.4.10. 3–(2-(6-Bromo-2-oxo-2H-chromen-3-yl)-2-oxoethyl)-5–(4-methoxybenzylidene)thiazolidine-2,4-dione 10j

Grey crystals (yield, 72%); m. p. = 245–247 °C; ^1^H NMR (400 MHz, DMSO-d_6_) *δ* 8.60 (s, 1H, ArH), 8.21 (d, *J* = 2.4 Hz, 1H, ArH), 7.87 (d, *J* = 8.0 Hz, 1H, ArH), 7.73 (s, 1H, –CH=), 7.54 (d, *J* = 8.8 Hz, 2H, ArH), 7.43 (d, *J* = 8.0 Hz, 1H, ArH), 7.08 (d, *J* = 8.8 Hz, 2H, ArH), 5.20 (s, 2H, –N–CH_2_–CO–), 3.82 (s, 3H, OCH_3_); Anal. Calcd. for C_22_H_14_BrNO_6_S (500.32): C, 52.81; H, 2.82; N, 2.80; Found: C, 53.04; H, 2.83; N, 2.80.

##### 2.1.4.11. 3–(2-(6-Bromo-2-oxo-2H-chromen-3-yl)-2-oxoethyl)-5–(2,5 -dimethoxybenzylidene) thiazolidine-2,4-dione 10k

Yellow crystals (yield, 78%); m. p. = 204–206 °C; ^1^H NMR (400 MHz, DMSO-d_6_) *δ* 8.59 (s, 1H, ArH), 8.21 (d, *J* = 2.4 Hz, 1H, ArH), 7.90 (s, 1H, –CH=), 7.87 (d, *J* = 8.0 Hz, 1H, ArH), 7.42 (d, *J* = 8.0 Hz, 1H, ArH), 7.08–7.07 (m, 2H, ArH), 6.91 (d, *J* = 8.0 Hz, 1H, ArH), 5.19 (s, 2H, –N–CH_2_–CO–), 3.83 (s, 3H, OCH_3_), 3.75 (s, 3H, OCH_3_); ^13 ^C NMR (101 MHz, TFA-deuterated) *δ* 162.46, 162.02, 161.74, 161.59, 161.15, 154.65, 152.12, 134.08, 122.06, 120.32, 118.49, 115.74, 115.68, 113.18, 112.86, 110.05, 56.32, 55.38. Anal. Calcd. for C_23_H_16_BrNO_7_S (530.35): C, 52.09; H, 3.04; N, 2.64; Found: C, 51.89; H, 3.07; N, 2.63.

##### 2.1.4.12. 3–(2-(6-Bromo-2-oxo-2H-chromen-3-yl)-2-oxoethyl)-5–(4- nitrobenzylidene)thiazolidine-2,4-dione 10 l

Yellow crystals (yield, 82%); m. p. = 229–231 °C; ^1^H NMR (400 MHz, DMSO-d_6_) *δ* 8.77 (s, 1H, ArH), 8.38 (s, 1H, ArH), 8.32 (d, *J* = 8.8 Hz, 2H, ArH), 7.93 (dd, *J* = 8.8, 2.0 Hz, 1H, ArH), 7.88 (s, 1H, –CH=), 7.84 (d, *J* = 8.8 Hz, 2H, ArH), 7.49 (d, *J* = 8.8 Hz, 1H, ArH), 5.13 (s, 2H, –N–CH_2_–CO–); ^13 ^C NMR (101 MHz, DMSO) *δ* 189.24, 168.05, 167.94, 166.89, 165.28, 158.81, 154.31, 148.23, 147.88, 139.95, 139.52, 131.65, 131.35, 129.28, 128.90, 125.63, 124.73, 123.59, 120.40, 117.06, 50.98. Anal. Calcd. for C_21_H_11_BrN_2_O_7_S (515.29): C, 48.95; H, 2.15; N, 5.44; Found: C, 49.11; H, 2.14; N, 5.48.

##### 2.1.4.13. 3–(2-(6-Bromo-2-oxo-2H-chromen-3-yl)-2-oxoethyl)-5–(2-chlorobenzylidene)thiazolidine-2,4-dione 10m

Yellow crystals (yield, 77%); m. p. = 192–194 °C; ^1^H NMR (400 MHz, DMSO-d_6_) *δ* 8.65 (s, 1H, ArH), 8.12 (s, 1H, ArH), 8.05 (d, *J* = 8.8 Hz, 1H, ArH), 7.75–7.51 (m, 6H, ArH), 5.64 (s, 2H, –N–CH_2_–CO–); ^13 ^C NMR (101 MHz, TFA-deuterated) *δ* 162.52, 162.09, 161.66, 161.22, 151.67, 139.52, 138.48, 135.27, 133.00, 132.66, 132.42, 130.36, 128.68, 127.03, 119.33, 118.51, 115.69, 112.88, 110.07. Anal. Calcd. for C_21_H_11_BrClNO_5_S (504.74): C, 49.97; H, 2.20; N, 2.78; Found: C, 50.13; H, 2.21; N, 2.80.

##### 2.1.4.14. 3–(2-(6-Bromo-2-oxo-2H-chromen-3-yl)-2-oxoethyl)-5–(2-bromobenzylidene)thiazolidine-2,4-dione 10n

Yellow crystals (yield, 72%); m. p. = 208–210 °C; ^1^H NMR (400 MHz, DMSO-d_6_) *δ* 8.61 (s, 1H, ArH), 8.22 (s, 1H, ArH), 7.87 (d, *J* = 8.8 Hz, 1H, ArH), 7.79 (s, 1H, –CH=), 7.51–7.59 (m, 3H, ArH), 7.43 (d, *J* = 8.8 Hz, 1H, ArH), 7.34 (t, *J* = 8.0 Hz, 1H, ArH), 5.14 (s, 2H, –N–CH_2_–CO–); ^13 ^C NMR (101 MHz, DMSO) *δ* 207.46, 158.94, 158.47, 154.43, 154.09, 146.11, 137.06, 134.21, 134.16, 133.92, 133.01, 131.75, 129.88, 129.61, 129.39, 129.23, 128.93, 120.54, 118.88, 116.83, 30.50. Anal. Calcd. for C_21_H_11_Br_2_NO_5_S (549.19): C, 45.93; H, 2.02; N, 2.55; Found: C, 46.09; H, 2.03; N, 2.53.

#### 2.1.5. General procedures for preparation of target coumarins 11a–d

5-(Thiophen-2-ylmethylene)thiazolidine-2,4-dione **7a** (15 mmol) and/or 5-((5-methylfuran-2-yl)methylene)thiazolidine-2,4-dione **7b** (15 mmol) was added to a hot stirred solution of 3-(bromoacetyl) coumarin derivatives **3a,b** (15 mmol) in DMF (10 ml), K_2_CO_3_ (15 mmol), KI (15 mmol), then the resulting mixture was stirred under reflux for 8h. The precipitates were collected by filtration, washed with water, dried and recrystalslized from hexane/ethanol to yield the final target compounds **11a-d.**

##### 2.1.5.1. 3–(2-Oxo-2–(2-oxo-2H-chromen-3-yl)ethyl)-5-(thiophen-2-ylmethylene)thiazolidine-2,4-dione 11a

Yellow crystals (yield, 83%); m. p. = 250–251 °C; ^1^H NMR (400 MHz, DMSO-d_6_) *δ* 9.13 (s, 1H, ArH), 8.44 (s, 1H, –CH=), 7.97–7.91 (m, 3H, ArH), 7.68–7.61 (m, 3H, ArH), 7.33 (t, *J* = 4.8 Hz, 1H, ArH), 5.62 (s, 2H, CH_2_, –N–CH_2_–CO–); ^13 ^C NMR (101 MHz, TFA-deuterated) δ 190.74, 162.45, 162.02, 161.58, 161.14, 153.33, 137.00, 136.28, 134.41, 131.79, 131.08, 128.63, 126.38, 119.93, 118.48, 115.67, 112.85, 110.04, 50.74. Anal. Calcd. for C_19_H_11_NO_5_S_2_ (397.42): C, 57.42; H, 2.79; N, 3.52; Found: C, 57.26; H, 2.78; N, 3.55.

##### 2.1.5.2. 5-((5-Methylfuran-2-yl)methylene)-3–(2-oxo-2–(2-oxo-2H- chromen-3-yl)ethyl)thiazolidine-2,4-dione 11b

Yellow crystals (yield, 70%); m. p. = 227–229 °C; ^1^H NMR (400 MHz, DMSO-d_6_) *δ* 9.12 (s, 1H, ArH), 7.96–7.90 (m, 3H, –CH = and ArH), 7.62–7.60 (m, 2H, ArH), 7.09 (d, *J* = 4.0 Hz, 1H, ArH), 6.40 (d, *J* = 4.0 Hz, 1H, ArH), 5.60 (s, 2H, CH_2_, –N–CH_2_–CO–), 2.54 (s, 3H, CH_3_); ^13 ^C NMR (101 MHz, TFA-deuterated) *δ* 162.45, 162.02, 161.57, 161.14, 153.32, 147.87, 136.96, 131.05, 126.35, 124.21, 118.47, 118.02, 116.58, 115.66, 112.84, 110.55, 110.03, 110.03, 50.50, 12.05; Anal. Calcd. for C_20_H_13_NO_6_S (395.39): C, 60.76; H, 3.31; N, 3.54; Found: C, 60.92; H, 3.34; N, 3.51.

##### 2.1.5.3. 3–(2-(6-Bromo-2-oxo-2H-chromen-3-yl)-2-oxoethyl)-5-(thiophen-2-ylmethylene)thiazolidine-2,4-dione 11c

Yellow crystals (yield, 76%); m. p. = 255–257 °C; ^1^H NMR (400 MHz, DMSO-d_6_) *δ* 8.60 (s, 1H, ArH), 8.27 (d, *J* = 2.4 Hz, 1H, ArH), 8.04 (s, 1H, –CH=), 7.99 (d, *J* = 5.2 Hz, 1H, ArH), 7.93 (dd, *J* = 8.8, 2.4 Hz, 1H, ArH), 7.66 (d, *J* = 8.0 Hz, 1H, ArH), 7.48 (d, *J* = 3.6 Hz, 1H, ArH), 7.27 (t, *J* = 3.6 Hz, 1H, ArH), 5.20 (s, 2H, –N–CH_2_–CO–); ^13 ^C NMR (101 MHz, DMSO) *δ* 189.40, 167.97, 167.84, 165.35, 158.79, 147.90, 146.11, 137.84, 137.41, 136.00, 134.92, 133.42, 129.39, 127.63, 125.40, 121.86, 120.42, 117.04, 50.93. Anal. Calcd. for C_19_H_10_BrNO_5_S_2_ (476.32): C, 47.91; H, 2.12; N, 2.94; Found: C, 47.79; H, 2.13; N, 2.96.

##### 2.1.5.4. 3–(2-(6-Bromo-2-oxo-2H-chromen-3-yl)-2-oxoethyl)-5-((5-methylfuran-2-yl)methylene)thiazolidine-2,4-dione 11d

Yellow crystals (yield, 79%); m. p. = 236–238 °C; ^1^H NMR (400 MHz, DMSO-d_6_) *δ* 8.60 (s, 1H, ArH), 8.22 (d, *J* = 2.4 Hz, 1H, ArH), 7.99 (dd, *J* = 8.8, 2.4 Hz, 1H, ArH), 7.74 (s, 1H, –CH=), 7.43 (d, *J* = 8.8 Hz, 1H, ArH), 6.99 (d, *J* = 4.0 Hz, 1H, ArH), 6.39 (d, *J* = 4.0 Hz, 1H, ArH), 5.17 (s, 2H, –N–CH_2_–CO–), 2.38 (s, 3H, CH_3_); ^13 ^C NMR (101 MHz, DMSO) *δ* 189.51, 169.37, 167.80, 157.74, 148.41, 147.87, 146.11, 137.80, 137.06, 133.40, 133.01, 122.00, 120.69, 119.25, 119.05, 118.80, 111.03, 110.72, 50.58, 14.22. Anal. Calcd. for C_20_H_12_BrNO_6_S (474.28): C, 50.65; H, 2.55; N, 2.95; Found: C, 50.52; H, 2.57; N, 2.98.

### 2.2. Biological evaluation

The experimental procedures for CA stopped-flow [29–31], MTT cell viability [32,33], cell cycle [34] and Annexin V-FITC/PI [35] assays are included in the Supporting Information.

## 3. Results and discussion

### 3.1. Chemistry

**Scheme 1.**
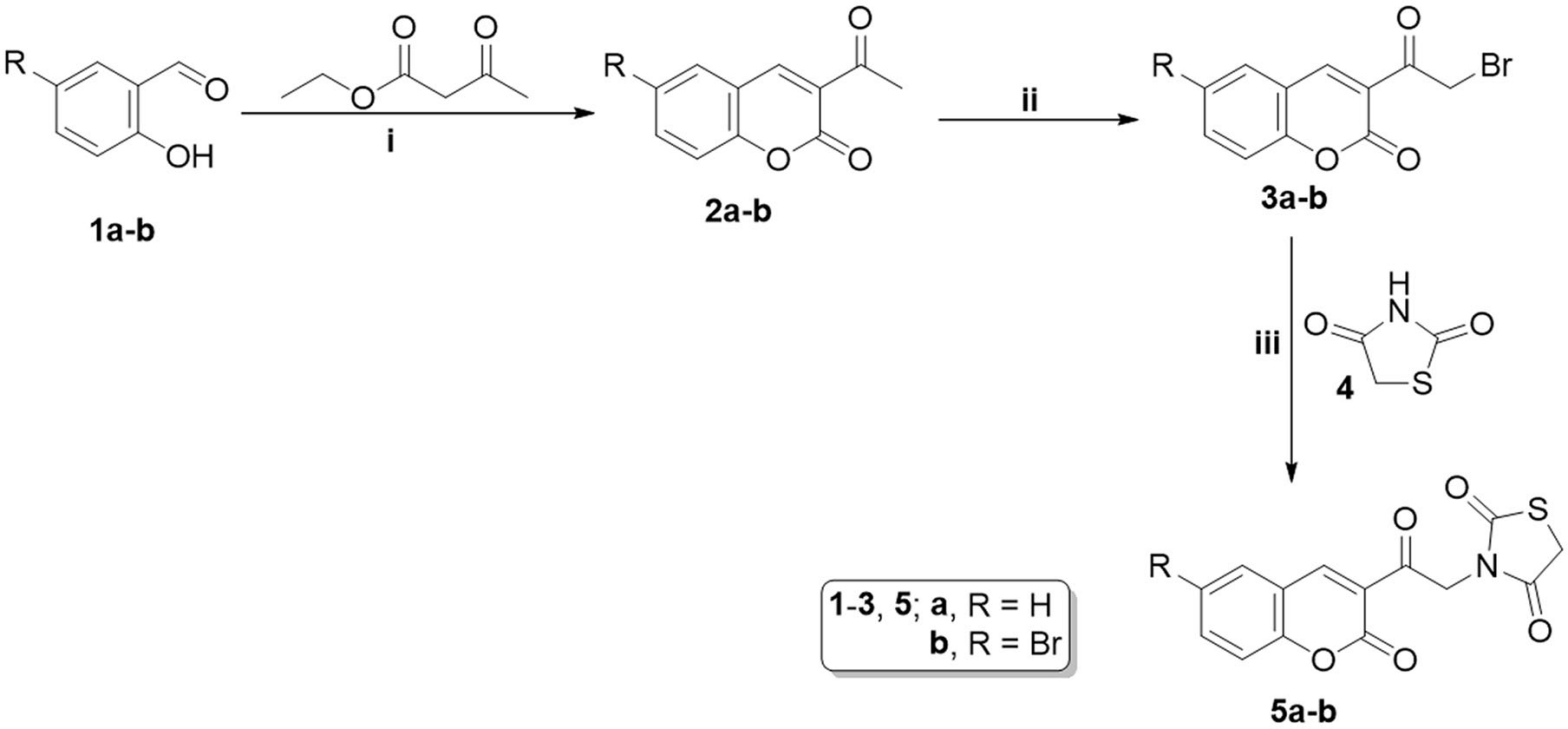
Reagents and conditions: (i) Abs. Ethanol, piperidine, reflux, 2 h.; (ii) bromine 99%, glacial acetic acid, r.t., 6 h.; (iii) anhydrous DMF, potassium carbonate, potassium iodide, heating on a water bath, 8 h.

**Scheme 2.**
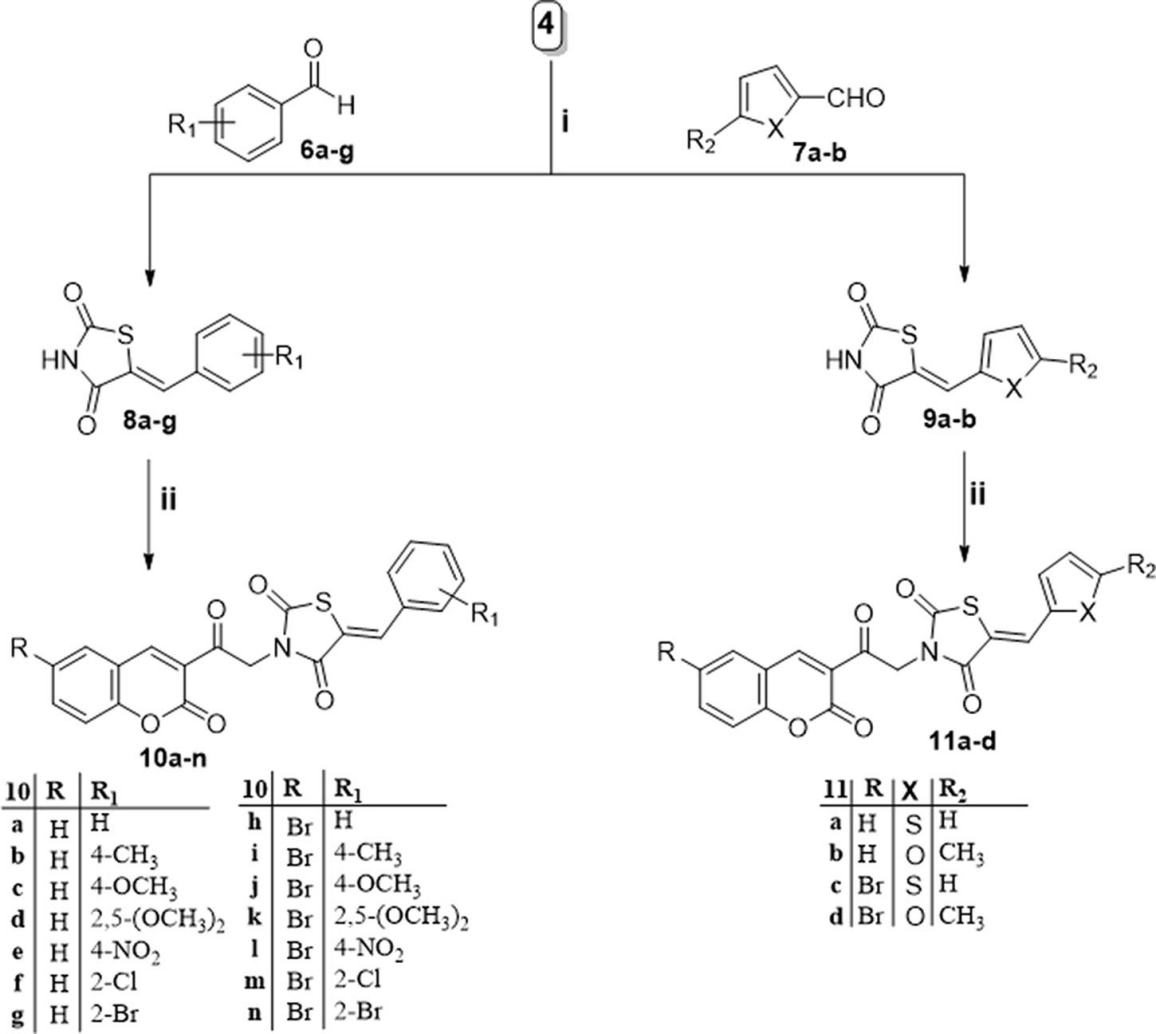
Reagents and conditions: (i) glacial acetic acid, reflux 3 h.; (ii) DMF, potassium carbonate, potassium iodide, reflux 8 h.

The proposed synthetic routes to obtain the target coumarins are depicted in Scheme 1 and Scheme 2. First, condensation of 2-hydroxybenzaldehydes **1a–b** with ethyl 3-oxobutanoate in refluxing absolute ethanol in the presence of a few drops of piperidine yielded 3-acetylcoumarins **2a–b**. These were subjected to bromination via reaction with Br_2_ in glacial acetic acid to yield the key 3-(bromoacetyl)coumarin intermediates **3a–b**, which were subsequently treated with thiazolidine-2,4-dione **4** in refluxing DMF using anhydrous K_2_CO_3_ as base and KI as a nucleophilic catalyst to afford 3–(2-oxo-2–(2-oxo-2*H*-chromen-3-yl)ethyl)thiazolidine-2,4-dione derivatives **5a–b** (Scheme 1).

Synthesis of compounds **8a–g** and **9a–b** (Scheme 2) was achieved *via* refluxing of thiazolidine-2,4-dione **4** with benzaldehyde derivatives **6a–g** and **7a–b** in glacial acetic acid and anhydrous sodium acetate. Treatment of **8a–g** and **9a–b** with the key intermediates **3a–b** in refluxing DMF using anhydrous K_2_CO_3_ and KI furnished the corresponding final targets 5-benzylidene-3–(2-oxo-2–(2-oxo-2H-chromen-3-yl)ethyl)thiazolidine-2,4-dione **10a–n** and **11a–d**, respectively. Proposed structures for the synthesised coumarins were in agreement with their various spectroscopic and analytical data.

### 3.2. Carbonic anhydrase inhibition

The inhibitory influence of all the synthesised coumarins **5a–b**, **10a–n** and **11a–d** was investigated against *h*CA I, II IX and XII isoforms using a stopped flow CO_2_ hydrase assay and a well-known *h*CAI, acetazolamide (**AAZ**) as control [36]. From the resulting inhibition constants (K_I_) shown in Table 1, certain structure activity relationship (SAR) can be inferred.

**Table 1.** Inhibition data for *h*CA I, II, IX and XII isoforms with 2,4-thiazolidinedione-tethered coumarins (**5a–b**, **10a–n** and **11a–d**) and **AAZ**. 
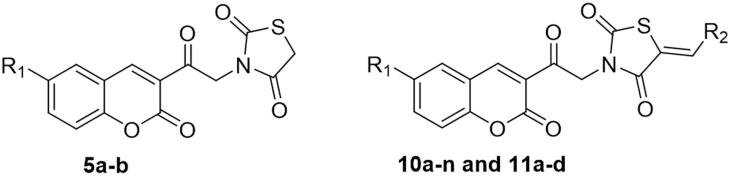

Cmpd	R_1_	R_2_	*K*_I_ (μM)^a,b^			
			CA I	CA II	CA IX	CA XII
**5a**	H	–	>100	>100	0.12	0.15
**5b**	Br	–	>100	>100	0.24	0.31
**10a**	H	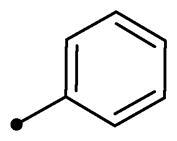	>100	>100	0.82	0.75
**10b**	H	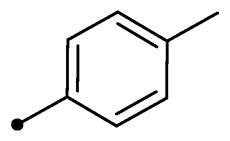	>100	>100	4.3	4.0
**10c**	H	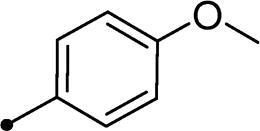	>100	>100	5.8	4.5
**10d**	H	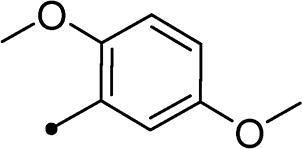	>100	>100	8.4	6.2
**10e**	H	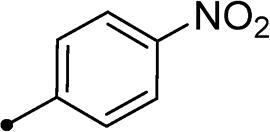	>100	>100	12.3	8.0
**10f**	H	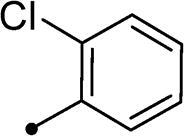	>100	>100	2.2	3.8
**10g**	H	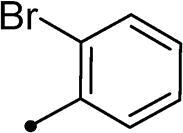	>100	>100	2.3	4.1
**10h**	Br	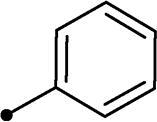	>100	>100	0.93	0.87
**10i**	Br	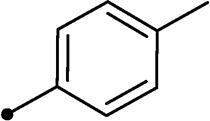	>100	>100	6.2	2.3
**10j**	Br	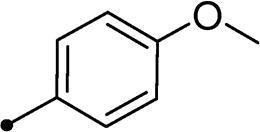	>100	>100	8.9	4.9
**10k**	Br	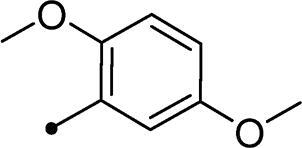	>100	>100	16.4	6.6
**10l**	Br	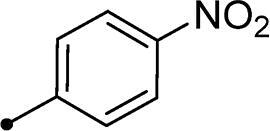	>100	>100	18.2	10.4
**10m**	Br	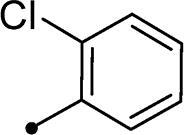	>100	>100	2.9	3.2
**10n**	Br	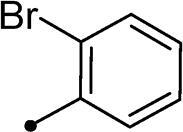	>100	>100	3.4	2.8
**11a**	H	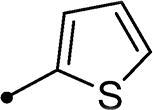	>100	>100	0.48	0.83
**11b**	H	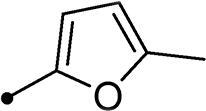	>100	>100	0.79	1.1
**11c**	Br	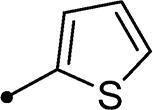	>100	>100	0.59	0.44
**11d**	Br	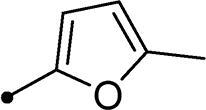	>100	>100	0.91	0.82
**AAZ**	–	–	250	12.5	25.0	5.7

^a^Mean from 3 different assays, by a stopped flow technique (errors were in the range of ± 5–10% of the reported values); ^b^incubation time of 6 h.

Coumarins **5a–b**, **10a–n** and **11a–d** are devoid of significant inhibition towards the off-target ubiquitous *h*CA I and the physiologically dominant *h*CA II (K_I_s > 100 µM) isoforms, Table 1. In contrast, these coumarins inhibited the cancer-related *h*CA IX with inhibition constants spanning a range between 0.12 and 18.2 µM, (Table 1). Regarding the unsubstituted thiazolidinedione-bearing coumarins **5a–b**, the absence of substitution at 6-position of coumarin scaffold furnished the most effective *h*CA IX inhibitor in this work displaying K_I_ of 0.12 µM, whereas, the 6-bromination decreased the *h*CA IX inhibitory power 2-folds (K_I_ = 0.24 µM).

In the context of *h*CA IX inhibition constants of the benzylidene counterparts **10a–n**, it was found that 6-unsubsituted coumarins **10a–g** showed effective inhibition (K_I_s ranged from 0.82 to 12.3 µM) compared to the 6-bromo analogues **10h–n** (K_I_s spanned between 0.93 and 18.2 µM). In the term of coumarins with the benzylidene moiety, **10a–g**, it is worth stressing that appending an unsubstituted aryl ring to the thiazolidinedione moiety **10a** provided the most effective *h*CA IX inhibitor within this series (K_I_ = 0.82 µM). Indeed, the incorporation of *ortho*-chloro or *ortho*-bromophenyl (**10f** and **10 g**, respectively) decreased the inhibition constants to low micromolar values (K_I_s = 2.2 and 2.3 µM, respectively). Regrettably, the remaining phenyl substitution pattern were similarly poorer *h*CA IX inhibitors with K_I_s equalling 4.3 µM (*p*-methyl, **10b**), 5.8 µM (*p*-methoxy, **10c**), 8.4 µM (2,5-dimethoxy, **10d**), 12.3 µM (*p*-nitro, **10e**).

In a similar fashion, the appending of unsubstituted aryl ring to the 6-bromocoumarins **10h–n** produced the most potent *h*CA IX inhibitor within this series (**10h**; K_I_ = 0.93 µM), whereas *ortho* chlorination or bromination reduced the inhibition constants to low micromolar values (**10m** and **10n**; K_I_s = 2.9 and 3.4 µM, respectively). Similar to the 6-unsubstituted coumarins **10a-g**, it was noted that in the 6-bromocoumarin series, inclusion of other phenyl substituents(compounds**10h–n)** lowered the *h*CA IX inhibition constants showing K_I_s in the range 6.2–18.2 µM. Superiorly, the replacement of six-membered phenyl with five-membered 2-thienyl ring potentially elevated the inhibition constants for both 6-unsubstituted and 6-bromocoumarins (**11a** and **11c**; K_I_s = 0.48 and 0.59 µM, respectively) compared to their phenyl counterparts (**10a** and **10h**; K_I_s = 0.82 and 0.93 µM, respectively). Notably, utilising 2-furyl functionality in place of phenyl/thienyl group did not result in significant change in potency (**11b** and **11d**; K_I_s = 0.79 and 0.91 µM, respectively).

Collectively, the deduced SAR for *h*CA IX inhibition suggests that applying of unsubstituted thiazolidinedione (**5a**–**b**) is more favoured than their substituted analogues (**10a–n** and **11a–d**) affording the most potent *h*CA IX inhibitors in this study. Furthermore, the lack of substitution at 6-position of coumarin (**5a**, **10a–g** and **11a–b**) is more advantageous for such type of activity relative to 6-bromo counterparts (**5b**, **10h–n** and **11c–d**). Additionally, appending of unsubstituted phenyl ring (**10a**, **10h**) is the most beneficial pattern within all tested benzylidene counterparts (**10a–n**), while replacement of phenyl with 2-thienyl moiety gave the most potent *h*CA IX inhibitors (**11a** and **11c**) within all arylidene derivatives (**10a–n** and **11a–d**).

Finally, the inhibition profiles (Table 1) revealed that the cancer-related *h*CA XII isoform was inhibited by the coumarins **5a–b**, **10a–n** and **11a–d** displaying a range of inhibition constants from submicromolar level to low micromolar values (K_I_s ranged from 0.15 to 10.4 µM). The unsubstituted thiazolidinedione-bearing coumarins **5a-b** emerged as the most effective *h*CA XII inhibitors, with submicromolar inhibition constants (**5a**; K_I_ = 0.15 µM and **5b**; K_I_ = 0.31 µM).

It should be pointed out that bromination at 6-position of coumarin **5b** led to 2-fold diminished inhibition for *h*CA XII relative to the unsubstituted analogue **5a**, in a similar manner observed in the SAR for *h*CA IX inhibition, Table 1. Concerning the benzylidene derivatives **10a–n**, it was observed that appending a phenyl to thiazolidinedione moiety resulted in the most potent *h*CA XII inhibitors (at submicromolar level) within this series (**10a**; K_I_ = 0.75 µM and **10h**; K_I_ = 0.87 µM). The incorporation of different substituents to the phenyl group reduced the inhibitory potential affording K_I_s spanning between 2.3 and 10.4 µM. Furthermore, the absence of substitution at 6-position of coumarin is beneficial for inhibition (**10a**; K_I_ = 0.75 µM), whereas 6-bromination decreased the activity (**10h**; K_I_ = 0.87 µM), Table 1. It was noted that replacement of the phenyl group (**10a**; K_I_ = 0.75 µM and **10h**; K_I_ = 0.87 µM) with 2-thienyl or 2-furyl functionalities reduced K_I_s for the 6-unsubstituted coumarins (**11a**; K_I_ = 0.83 µM and **11b**; K_I_ = 1.1 µM, respectively), while raised K_I_s for the 6-bromocoumarins (**11c**; K_I_ = 0.44 µM and **11d**; K_I_ = 0.82 µM, respectively). This is unlike the pattern in *h*CA IX inhibition profile and points to a potential future avenue of exploration towards selectivity of *h*CA XII over *h*CA IX.

To summarise, the elicited SAR highlighted that unsubstituted thiazolidinedione derivatives (**5a**–**b**) exerted more superior potency relative to their substituted counterparts (**10a–n** and **11a–d**) resulting in the most potent *h*CA XII inhibitors in this work (**5a**-**b**). Moreover, within all benzylidene derivatives **10a–n**, the unsubstituted phenyl counterparts exerted the best inhibition profiles (**10a** and **10h**), however the replacement of phenyl with 2-thienyl or 2-furyl along with 6-bromination at coumarin scaffold potentiated the inhibitory impact of compounds (**11c** and **11d**). Overall, the herein reported coumarins emerge as selective inhibitors towards the tumour-related *h*CA IX and XII over the off-target *h*CA I and II that suggests their use as promising candidates for the development of more potent, selective *h*CA IX and XII inhibitors as anticancer agents.

### 3.3. Anticancer activity

#### 3.3.1. *In vitro* antiproliferative activity against MCF-7 breast cancer cell line

The antiproliferative action of the most potent and selective *h*CA IX/XII inhibitors **10a**, **10h** and **11a–d** was assessed against MCF-7 breast cancer cell line, since the overexpression of *h*CA IX is well-reported to be associated with poor prognosis of breast cancer [37] and the cell line has been previously used as a model in CA medicinal chemistry investigations. The antiproliferative potential was investigated using MTT assay [38] under hypoxic conditions employing staurosporine as a reference anticancer drug. The results are presented in Table 2 as median inhibitory concentration (IC_50_) which denotes the concentration of the tested drug required to produce 50% growth inhibition of the cancer cell compared to the negative control.

**Table 2.** Anti-proliferative activities of 2,4-thiazolidinedione-tethered coumarins **10a**, **10 h** and **11a–c** against MCF-7 cell line.

Compound	IC_50_ (μM)^a^ (MCF-7)
**10a**	3.13 ± 0.18
**10h**	11.1 ± 0.65
**11a**	0.48 ± 0.03
**11b**	4.14 ± 0.24
**11c**	9.56 ± 0.56
**11d**	1.65 ± 0.1
Staurosporine	2.44 ± 0.14

^a^IC_50_ values are the mean ± SD of three experiments.

Investigation of the antiproliferative effects towards MCF-7 breast cancer cell line confirmed that the tested coumarins **10a**, **10h** and **11a–c** exhibited moderate to excellent growth inhibitory influence (IC_50_ ranged between 0.48 and 11.1 µM). Of special interest, the 2-thienyl-bearing 6-unsubstitued coumarin **11a**, that displayed potent *h*CA IX/XII inhibition at submicromolar level, exerted excellent antiproliferative action at submicromolar value (IC_50_ equals 0.48 µM). Likewise, the other tested coumarins **10a**, **10h**, **11b–d** demonstrated moderate growth inhibitory action with IC_50_ values equal 3.13, 11.1, 4.14, 9.56 and 1.65 µM, respectively compared to staurosporine as reference drug (IC_50_ = 2.44 µM), Table 2.

#### 3.3.2. Cell cycle analysis

**Figure 2.**
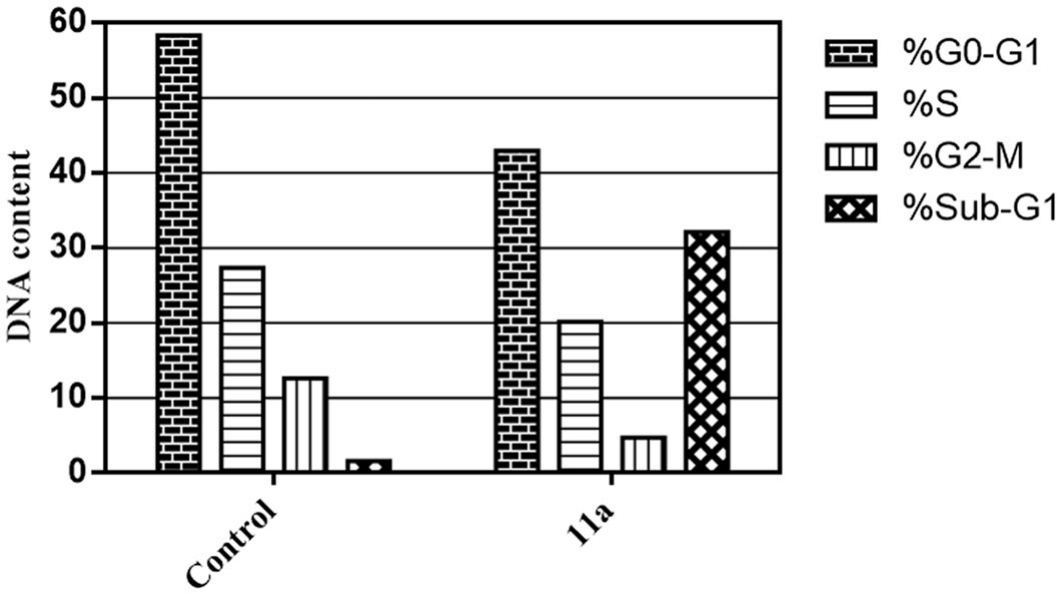
Impact of the tested coumarin **11a** on the progression of cell cycle of MCF-7 cells.

The influence of 2-thienyl-bearing 6-unsubstitued coumarin **11a** on the cell cycle progression was investigated by flow cytometric in MCF-7 breast cancer cells, at 24h following treatment at its IC_50_ value (0.48 ± 0.03 µM), Figure 2.

As illustrated in Figure 2, the flow cytometric results showed that the exposure of MCF-7 breast cancer cells to compound **11a** gave rise to a significant rise in the cell populations at Sub-G_1,_ which increased by 19.7 folds with concomitant decrease in G_2_-M phase by 2.6 folds compared to the control, in addition to decline in cell populations within S and G_0_-G_1_ phases. This observation strongly suggests coumarin **11a** induces apoptosis in MCF-7 cells.

#### 3.3.3. Annexin V-FITC/propidium iodide (AV/PI) apoptosis assay

**Figure 3.**
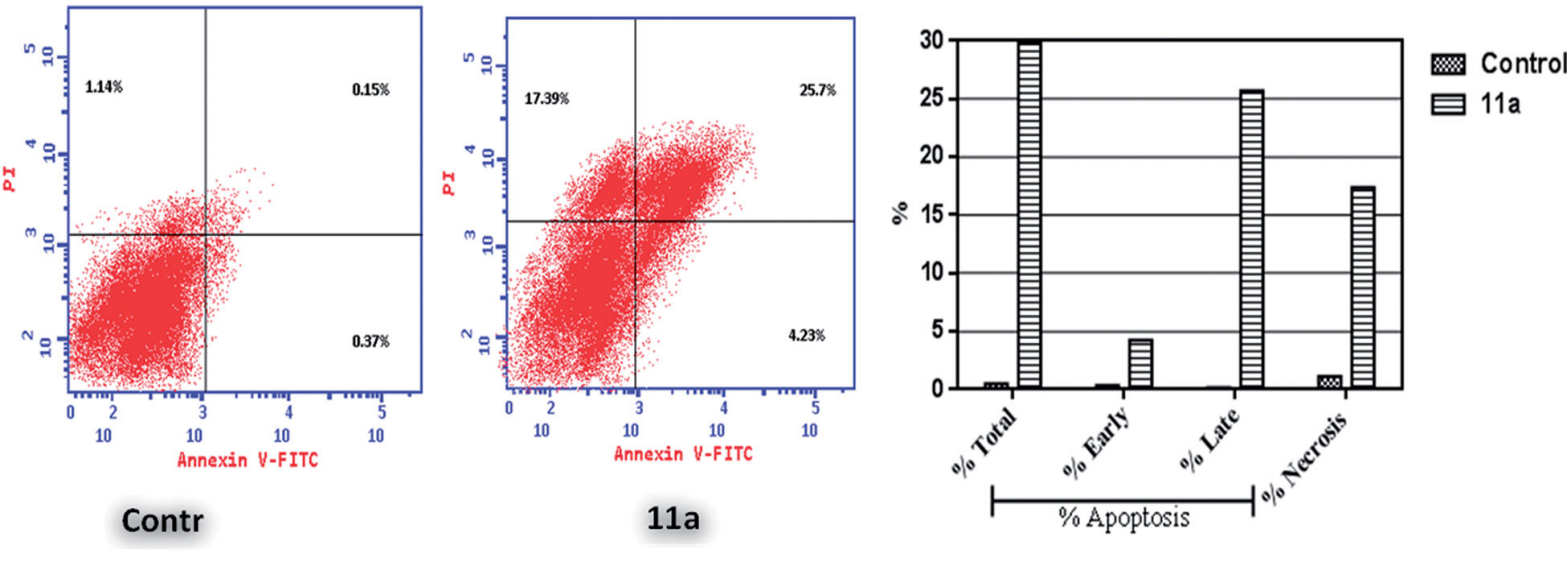
Effect of coumarin **11a** on the percentage of AV positive staining in breast MCF-7 cells.

Annexin V-FITC/propidium iodide (AnxV/PI) dual staining assay was employed to confirm the potential apoptotic impact of coumarin **11a** on early and late apoptosis percentages in MCF-7 breast cancer cells (Figure 3 and Table 3). This flow cytometric analysis highlighted that compound **11a** was able to induce apoptosis in MCF-7 cells as indicated by the significant elevation in the percentage of annexin V-FITC-stained apoptotic cells including early apoptosis (Figure 3, lower right) from 0.37 to 4.23% and late apoptosis, Figure 3, upper right) from 0.15 to 25.7%. This represents 57 folds total increase relative to the control in apoptotic cells.

**Table 3.** Distribution of AV-FITC/PI positive stained apoptotic MCF-7 cells.

Comp.	Apoptosis			Necrosis
	Total	Early	Late	
**11a**	29.93	4.23	25.7	17.39
Control	0.52	0.37	0.15	1.14

## 4. Conclusions

In this study, different 2,4-thiazolidinedione-tethered coumarins **5a–b**, **10a–n** and **11a–d** have been synthesised and evaluated for their inhibitory action against the cancer-associated *h*CAs IX and XII, in addition to the physiologically dominant *h*CAs I and II, in order to explore their selectivity. Interestingly, none of the coumarins had any inhibitory effect on off-target *h*CA I and II isoforms. Unsubstituted phenyl-bearing coumarins **10a**, **10h**, and 2-thienyl/furyl-bearing coumarins **11a–c** exhibited the best *h*CA IX (K_I_s between 0.48 and 0.93 µM) and *h*CA XII (K_I_s between 0.44 and 1.1 µM) inhibitory actions. Coumarins **10a**, **10h** and **11a–c** were subjected to an *in vitro* antiproliferative assay, and then the most potent antiproliferative agent **11a** was tested to explore its impact on the cell cycle phases and apoptosis in MCF-7 breast cancer cells furnishing more insights on the potential anticancer activity of such compounds.

## Supplementary Material

Supplemental Material
