# Supplementary material for: Discovery of 2,4-thiazolidinedione-tethered coumarins as novel selective inhibitors for carbonic anhydrase IX and XII isoforms
Source: J Enzyme Inhib Med Chem. 2022 Jan 7;37(1):531–41. doi: 10.1080/14756366.2021.2024528 (PMC8745369; doi:10.1080/14756366.2021.2024528)
Supplement: Supplemental Material [file IENZ_A_2024528_SM4083.pdf]

Supporting Information

Discovery of 2,4-thiazolidinedione-tethered coumarins as novel selective inhibitors for carbonic anhydrase IX and XII isoforms

Wagdy M. Eldehna[*], Mohammed S. Taghour, Tarfah Al-Warhi, Alessio Nocentini, Mostafa M. Elbadawi, Hazem A. Mahdy, Mohamed A. Abdelrahman, Ohoud J. Alotaibi[c], Nada Aljaeed, Diaaeldin M. Elimam, Kamyar Afarinkia, Hatem A. Abdel-Aziz, Claudiu T. Supuran[*]

Tables of Contents

1. **Carbonic Anhydrase Inhibition Assay** 2

2. **Anti-proliferative Activity** 2

3. **Cell Cycle Analysis** 3

4. **AnnexinV-FITC/PI Apoptosis Assay** 3

1. Carbonic anhydrase inhibition assay

The carbonic anhydrase catalyzed CO_2 hydration actions for all coumarin-based derivatives reported in this study have assayed utilizing an instrument of Applied Photophysics stopped-flow. The enzymes are recombinant proteins prepared in our lab. Phenol red (at a concentration of 0.2 mM) has been used as indicator, working at the absorbance maximum of 557 nm, with 20 mM Hepes (pH 7.5) as buffer, and 20 mM Na_2SO_4 (for maintaining constant the ionic strength), following the initial rates of the CA-catalyzed CO_2 hydration reaction for a period of 10-100 s. The CO_2 concentrations ranged from 1.7 to 17 mM for the determination of the kinetic parameters and inhibition constants. For each inhibitor at least six traces of the initial 5-10% of the reaction have been used for determining the initial velocity. The uncatalyzed rates were determined in the same manner and subtracted from the total observed rates. Stock solutions of inhibitor (0.1 mM) were prepared in distilled-deionized water and dilutions up to 0.01 nM were done thereafter with the assay buffer. Inhibitor and enzyme solutions were preincubated together for 6 hrs. at room temperature prior to assay, in order to allow for the formation of the E-I complex. The inhibition constants were obtained by non-linear least-squares methods using PRISM 3 and the Cheng-Prusoff equation, and represent the mean from at least three different determinations.

2. Anti-proliferative action toward human breast cell line

The examined human breast cancer cell line (MCF-7) has been obtained from American Type Culture Collection (ATCC). Cells line was maintained as monolayers in Dulbecco's Modified Eagle's Medium (DMEM) supplemented with 10% FBS, 2 mM L-glutamine, 100 U/ml penicillin and 100µg/ml streptomycin sulfate. Cobalt (II) chloride ($CoCl_2$) was utilized as an inducer of HIF-1α to furnish a chemically-induced hypoxia. Cells were sub-cultured with trypsine /EDTA solution, counted with haemocytometer and plated onto 96-well plates (5000 cells/well) and left overnight to form a semi-confluent monolayer. Cell monolayers were treated in quadrates with vehicle (DMSO, 0.1% v/v), test samples (**10a, 10h and 11a-c**) or Staurosporine as positive control for an exposure time of 48 h. At the end of exposure, MTT solution in PBS (5 mg/ml) was then added to all well including no cell blank and left to incubate for 90 min. The formation of formazan crystals were visually confirmed using phase contract microscopy. DMSO (100 µl/well) was added to dissolve the formazan crystals with shaking for 10 min after which the absorbance was read at

590 nm against no cell blanks on a FLuo Star Optima microplate reader (BMG technologies, Germany). Cell proliferation was calculated comparing the OD values of the DMSO control wells and those of the sample represented as % proliferation to the control. Dose-response experiment was performed on samples producing > or =50% loss of cell proliferation using five serial 2-fold dilutions (50, 25, 12.5, 6.25 and 3.125 µM) of the sample. IC_{50} values (concentration of sample causing 50% loss of cell proliferation of the vehicle control) were calculated using non-linear regression curve fitting of the dose response plots on GraphPad Prism V.6.0 software.

3. Cell Cycle Analysis

Breast cancer MCF-7 cells were treated with coumarin **11a** for 24 h (at its IC_{50} concentration), and then cells were washed twice with ice-cold phosphate buffered saline (PBS). Subsequently, the treated cells were collected by centrifugation, fixed in ice-cold 70% (*v*/*v*) ethanol, washed with PBS, re-suspended with 100 µg/mL RNase, stained with 40 µg/mL PI, and analyzed by flow cytometry using FACS Calibur (Becton Dickinson, BD, Franklin Lakes, NJ, USA). The cell cycle distributions were calculated using CellQuest software 5.1 (Becton Dickinson).

4. Annexin V-FITC Apoptosis Assay

Phosphatidylserine externalization was assayed using Annexin V-FITC/PI apoptosis detection kit (BD Biosciences, USA) according to the manufacturer's instructions. Breast cancer MCF-7 cells were cultured to a monolayer then treated with coumarin **11a** at its IC_{50} concentration. Briefly, cells were then harvested *via* trypsinization, and rinsed twice in PBS followed by binding buffer. Moreover, cells were re-suspended in 100 µL of binding buffer with the addition of 1 µL of FITC-Annexin V followed by an incubation period of 30 min at 4 °C. Cells were then rinsed in binding buffer and resuspended in 150 µL of binding buffer with the addition of 1 µL of DAPI (1 µg/µL in PBS). Cells were then analyzed using the flow cytometer BD FACS Canto II and the results were interpreted with FlowJo7.6.4 software (Tree Star, Ashland, OR, USA).
